# Judgment, shame, and coercion: the criminal legal system and reproductive autonomy

**DOI:** 10.1186/s40352-024-00259-8

**Published:** 2024-02-16

**Authors:** Ginny Garcia-Alexander, Melissa Thompson

**Affiliations:** 1 Department of Sociology, University of Texas at San Antonio, One UTSA Circle, San Antonio, TX 78249 USA; 2 Department of Sociology, Portland State University, P.O. Box 751, Portland, OR 97201 USA

**Keywords:** Arrest, Contraception, Sterilization, Reproductive autonomy, Criminal legal system

## Abstract

**Background:**

A growing body of research has called attention to limitations to reproductive autonomy in both women who are socially disadvantaged and in those who have had contact with the criminal legal (CL) system. However, it is unclear whether CL system contact influences contraceptive use patterns and how these processes unfold. We utilize a mixed-methods approach to investigate whether history of arrest is associated with receipt of contraceptive counseling, use of long-term contraception, sterilization, and subsequent desire for reversal of sterilization. We further consider how agents in and around the CL system may influence women’s reproductive decisions and outcomes (856 survey respondents; 10 interviewees).

**Results:**

We observe that women who have been arrested more commonly report receipt of contraceptive counseling and sterilization. They are also significantly more likely to want their sterilization reversed. Our in-depth interviews suggest that women with CL contact experience considerable shame, and in some cases, coercion to limit fertility from various agents in and outside the criminal legal system including medical providers, Parole/Probation Officers (POs), guards, and family members.

**Conclusions:**

Our findings suggest the need for ongoing attention to how exposure to this system may promote uneven use of certain forms of contraception and dissatisfaction, i.e., desire for reversal of sterilization, among these women. Findings further suggest that de-emphasizing the CL system as a means through which to address reproductive needs should be considered.

## Highlights

· We use a mixed-methods approach to establish linkages between arrest history and contraceptive use.

· Arrest is an important point of contact with the criminal legal system that contributes to fertility limitation in exposed women.

· Women who have been arrested are more likely to want a sterilization procedure reversed.

· Women with arrest histories experience significant shaming regarding their fertility desires and outcomes from various agents in and around the criminal legal system.

## Background

The criminal legal (CL) setting is a site with significant potential to limit reproductive autonomy via direct and indirect mechanisms (Pan et al., 2021; Winters & McLaughlin, 2019). Historically, the U.S. CL system has regulated women’s reproductive choices directly via policies supporting coercive or forcible sterilizations (Kluchin, 2009; Schoen, 2005; Solinger, 2005). This has persisted into the present as coercive and forcible sterilizations among CL-involved women continue to be documented (Burke, 2015; McGreevy & Willon, 2013). Recent media stories have also brought attention to the CL system’s role in actively encouraging young women with arrest histories to use contraception, particularly long-term methods (ACLU, 2017). Indirectly, the idea that CL contact reduces one’s capacity for “fit” motherhood appears to be expressed regularly by various agents within the CL system, e.g., judges, attorneys, and probation officers (POs), who may engage in efforts at fertility limitation. This is likely a product of widely held assumptions that the incarcerated mother is a “bad mother” with a deeply flawed character. These women are intensely stigmatized and, “among the most marginalized women in society” (Aiello & McQueeny, 2016: p. 32). Thus, efforts to prevent or restrict the childbearing of CL-involved women are reflected by instances of prosecutors requiring sterilization in exchange for plea deals (Burke, 2015); judges offering reduced sentences if permanent or long-term contraception is used (Flavin, 2008; Perry, 2017); and parole or probation officers referring their clients to programs that offer cash incentives for using long-term contraception (Project Prevention, 2018; Flavin, 2008).

Further complicating this issue is that women who are socially disadvantaged, e.g., lower socioeconomic status, uninsured, racial/ethnic minorities, and/or unemployed, are disproportionately exposed to the CL system (Cowan, 2019; Rich et al., 2014; Shannon et al., 2017; Uggen et al., 2006; Western, 2006). Socially marginalized women experience reduced access to, and quality of care, and they have been found to be disproportionately targeted for the promotion of long-term methods of contraception (Higgins et al., 2016). Studies have observed that low-income and women of color are more commonly advised by physicians to limit their childbearing (Downing et al., 2007) or felt coerced to use contraception (Becker & Tsui, 2008; Yee & Simon, 2011). These characteristics have also been associated with overuse of long-term and permanent methods of contraception (Berttoti, 2009; Garcia-Alexander et al., 2019; Malat, 2000; Shreffler et al., 2015).

Women’s exposure to the CL system has increased exponentially over time (UCR, 2022; The Sentencing Project, 2021), which is important as this contact coincides with peak reproductive years (ACOG, 2021). Recently, growing awareness of power differentials between actors throughout the CL system and the potential for abuse has led to calls for explicit attention to whether women’s reproductive autonomy is preserved in these settings (Sufrin et al., 2015). There has also been explicit concern about how the earliest stage of the CL system (arrest) has been understudied despite the relatively large number of girls and women—especially women of color—who are impacted at this CL stage, but not likely to experience later CL stages (Gartner, 2011; Spinney et al., 2018). Vulnerable populations are disproportionately subject to arrest; and the characteristics that make women vulnerable (e.g., ethnic minority status, low SES, low education) are linked with higher usage of sterilization and other methods of contraception that are argued to remove or significantly reduce user agency. These patterns raise questions about the extent to which differences in contraceptive use patterns are reflective of women’s preferences, constraints, or some form of coercion. It is also unknown how CL system involvement may lead women to devalue their own childbearing, which may further inhibit desired reproductive outcomes.

We employ a mixed-methods approach to investigate the role of CL system involvement in shaping women’s reproductive capacity and advance the literature in this area in several important ways. First, we conduct quantitative analyses using nationally representative data to establish how arrest patterns long-term and permanent contraceptive use and subsequent desire for reversal of sterilization procedure. Second, we supplement our quantitative analyses with in-depth interviews of women with arrest histories to shed light on the mechanisms and actors that may contribute to a lack of reproductive autonomy in this population.

Our research questions therefore ask: (1) does CL system contact (arrest) shape women’s reproductive capacity? Specifically, (1a) is receipt of contraceptive counseling, long-term contraceptive use, receipt of sterilization, and desire for sterilization reversal, affected by CL system contact? and (2) how does contact with the CL system contribute to reduced reproductive autonomy?

## Methods

### Surveys

Our data are taken from the 2021 Crime, Health, and Politics Survey (CHAPS). CHAPS is based on a national probability sample of 1,771 community-dwelling adults aged 18 and over living in the United States. Respondents were sampled from the National Opinion Research Center’s (NORC) AmeriSpeak© panel, which is representative of households from all 50 states and the District of Columbia (https://amerispeak.norc.org/Documents/Research/AmeriSpeak%20Technical%20Overview%202019%2002%2018.pdf). Sampled respondents were invited to complete the online survey in English between May 10, 2021 and June 1, 2021. The data collection process yielded a survey completion rate of 30.7% and a weighted cumulative response rate of 4.4%. The multistage probability sample resulted in a margin of error of ± 3.23% and an average design effect of 1.92. The median self-administered web-based survey lasted approximately 25 min. All respondents were offered the cash equivalent of $8.00 for completing the survey. The survey was reviewed and approved by the institutional review board at NORC and one other university review board. Informed consent was obtained from all participants. The primary purpose of CHAPS is to document the social causes and social consequences of various indicators of health and well-being in the United States during the coronavirus (COVID-19) pandemic.

### Qualitative Interviews

In addition to the CHAPS survey, we conducted a supplementary qualitative semi-structured interview project, recruiting CL system-involved women (those with at least one previous arrest) living in rural, suburban, and urban counties in Oregon. Potential respondents were eligible for the study if they self-reported being a woman with at least one prior arrest and were at least 18 years of age at the time of the interview. No other selection criteria were imposed.

Qualitative interviews were chosen for this supplemental data collection because they allow us to seek and develop knowledge from marginalized populations whose experiences are not easily predetermined or quantifiable (Ferraro & Moe, 2003, p. 15). The resulting data allow us to interpret and explain some of the results that we find through the quantitative survey data. These qualitative interviews were designed to supplement and expand on the quantitative data—providing context, explanations, and experiences that would otherwise be impossible to gain through sole reliance on quantitative data.

Our interview project was reviewed and approved by one university’s institutional review board and informed consent was obtained from all interviewees. Our recruitment efforts for these interviews sought a racially diverse sample and focused on what affects reproductive options from the perspective of interview subjects. Because this data collection effort took place during the height of COVID-19, recruitment efforts took place online or via the mail, and interviews were conducted over the phone. We collaborated with county Departments of Community Justice to make potential participations aware of this data collection effort. We also sought to recruit through organizations serving formerly incarcerated individuals. These organizations included Oxford Houses, homeless shelters, and faith-based organizations.

Over a 12-month period—from April 2020 through March 2021—recruitment efforts resulted in 10 completed interviews. About half of the interview subjects were referred by a Parole or Probation Officer (PO), although POs were not told whether their clients were actually interviewed (POs shared fliers—which provided information about the research project—with their clients; it was up to the client to decide whether to reach out to the research team, and we did not inform POs which clients did, or did not, follow up). The actual interviews were conducted separately from POs and PO offices (interviews were conducted over the phone, and respondents were asked to be wherever they were comfortable talking about private information). The remaining participants were found through our outreach to Oxford Houses throughout the state of Oregon. Fliers were mailed to Oxford Houses requesting that eligible participants contact the research team. It was impossible for the research team to know who received the flier—either from POs, or at Oxford Houses—until the interested party contacted us, minimizing the potential for interview subjects to experience coercion or undue pressure from the research team. The recruitment flier indicated that interviews could be conducted in English or Spanish, although all 10 completed interviews were conducted in English. Two interviewers conducted these interviews. Respondents received $30 as an incentive for completing the interview. All phone interviews were recorded with a digital audio recorder, and these recordings were transcribed for analysis.

While we did not seek to verify respondent’s arrest histories, or any other information reported by our respondents, we do believe that the respondents were honest in their reports—especially since they were willing to disclose sensitive, difficult, and potentially stigmatizing experiences. One respondent reported a very high number of previous arrests (240, reported by Kaitlyn), but we believe this, like all other self-reported information, was an honest estimate since the number and frequency of arrests is commonly discussed with Parole Officers (Kaitlyn was on parole at the time of the interview). Moreover, Kaitlyn had significant CLS experience spanning a twenty-year period, and while the exact number of arrests was likely an estimate, we do believe that it was a reasonable one. In contrast, one respondent (Carla) had only one previous arrest, and another (Saige) had only two prior arrests. While we might expect that the life experiences of a woman arrested one time—compared to one arrested over 200 times—might be considerably different, we report on the experiences of both extremes below. Importantly, we observed consistencies in our key themes in women with more severe criminal legal histories versus those with comparatively light histories.

## Measures

### Surveys

#### Reproductive outcomes

We focus on four outcomes related to reproductive care (see Table 1 for descriptive statistics for all measures). Receipt of contraceptive counseling is based on the question: “Have you ever received counseling about birth control or other contraceptive methods?” (1 = yes, 0 = no). Long-term contraception represents the combined responses to questions asking whether women have ever used: injectables, implants, or intrauterine devices (0 = no; 1 = yes, one or more). We measure sterilization with the question: “Have you ever had your tubes tied, cut, removed, or any other operation that makes it impossible for you to have a baby?” (1 = yes, 0 = no). Finally, we use responses to, “As things look to you now, if your procedure could be reversed safely, would you want to have it reversed?” (1 = yes, 0 = no).

**Table 1:** Weighted characteristics by arrest among female respondents, CHAPS 2021 (*N* = 856)

**Characteristic**	*N*	Never Arrested (%)	Arrested (%)	*p*
**All**	856	90.42	9.58	
**Contraceptive Counseling**				*.008*
No	256	32.67	21.29	
Yes	600	67.33	78.71	
**Long-Term Method (Injectable, Implant, IUD)**				*.067*
None	635	74.87	66.25	
One or More	221	25.16	33.75	
**Sterilization**				*.040*
No	577	69.85	57.31	
Yes	279	30.15	42.69	
**Desires Sterilization Reversal (*n* = 277)**				*.000*
No	240	92.22	48.85	
Yes	37	7.78	51.15	
**Age** (Mean (SE))	50.45 (.57)	48.83 (.94)	43.77 (2.26)	*.039*
**Race**				*.687*
NH White	591	63.31	66.89	
NH Black	98	12.80	14.92	
Hispanic	131	17.05	11.02	
NH Other	36	6.84	7.17	
**Education**				*.000*
No Degree	535	63.01	86.87	
College Degree +	321	36.99	13.13	
**Income**				*.000*
Less than $100,000	673	78.25	94.18	
$100,000 or more	183	21.75	5.82	
**Marital Status**				*.000*
Not Married	406	47.60	71.85	
Married	450	52.40	28.15	
**Type of Insurance**				*.036*
Private	404	48.50	32.03	
Government	335	37.65	55.33	
Other	75	9.21	6.09	
None	42	4.65	6.55	
**Desired No. Children** (Mean (SE))	2.20 (.06)	2.19 (.08)	2.83 (.24)	*.003*
**Number of Children** (Mean (SE))	1.68 (.05)	1.68 (.07)	1.69 (.17)	*.140*
**Sexual Partners (last 12 mos)** (Mean (SE))	0.94 (.07)	0.93 (.09)	1.37 (.32)	*.169*
**Region**				*.801*
South	292	38.36	39.40	
Other Region	564	61.64	60.60	

Means and percentages are weighted; N are unweighted

#### Arrest

Arrest history is measured based on responses to: “Have you ever been arrested for anything other than a minor traffic violation?” Responses included yes (1) or no (0).

#### Control variables

Covariates include race (1 = non-Hispanic White (reference), 2 = non-Hispanic Black, 3 = Hispanic, and 4 = non-Hispanic Other); Education (1 = four year college degree or higher; 0 = all other responses); Income (0 = less than $100,000, 1 = $100,000 or more), Marital Status (1 = married; 0 = else); Region (1 = South, 0 = else); Insurance (1 = private; 2 = government; 3 = other; 4 = none); Age (continuous); Number of Children (continuous); Desired Number of Children (continuous); and Number of Sexual Partners (continuous).

### Qualitative interviews

The qualitative interviews used a semi-structured format to create narratives of women’s experiences. This semi-structured script (see Appendix A for a complete list of the questions asked in the interview) sought to produce flexibility, allowing for tailored responses depending on the respondent’s own experiences with contraception, children (or, alternatively, a desire to *not* have children), and wide variations in criminal legal experiences. Interviews lasted approximately 30–40 min and were designed to measure individual history of contraception, desired fertility, contact with the criminal legal system, and how respondents have accessed and experienced women’s health care.

Because the qualitative interviews were intended to supplement, and expand on, the quantitative data, we sought to recruit interview subjects until we had identified evidence at least partially explaining and expanding on our key quantitative findings. We also kept in mind that we were seeking to interview a relatively hard-to-reach population (and during a pandemic). So, while we engaged in ongoing recruitment over the course of a year, we also opted to end recruitment once we identified evidence in the interviews speaking to the quantitative findings indicating that arrest history is associated with contraceptive counseling, sterilization, and a desire for reversal of sterilization. After finding evidence in the interviews speaking to these quantitative findings, we believe that we reached saturation with our 10 interviews. We further argue that, because we found considerable overlap in interviewee experience, and general consistency in terms of guilt and shaming experiences coming from contact with the CLS among the respondents, despite their wide array of criminal histories, we had little need to expand on the number of interviews conducted.

### Analyses

#### Surveys

Analyses were limited to women (*n* = 927). Listwise deletion of missing data resulted in an analytic sample of 856 (92% of women). Cross-tabulation and chi-square analyses or t-tests, were used to compare women who have been arrested to those who have not across all variables. Logistic regression analyses were used to examine the likelihood of various reproductive outcomes on the basis of arrest, while controlling for the effects of factors known to influence outcomes. Analyses were performed using STATA 17.0 (Statacorp, 2021).

### Qualitative interviews

After the interviews were transcribed, each transcript was reviewed and coded with a thematic analysis as described by Ryan and Bernard (2000), using a hybrid inductive/deductive approach. Initial coding was conducted by the second author, and identified themes were checked against the quantitative findings and by consulting with the first author. There were no issues with achieving consensus when identifying themes. These qualitative analyses were facilitated by using Atlas.ti software. The data were analyzed by thematic analytic coding, using both inductive and open coding, while also acknowledging the analysis that had already occurred using the quantitative survey data. First, we inductively analyzed interviews attending to salient perspectives of the respondents, particularly concerning their experiences with contraception, the criminal legal system, their perceived reproductive agency, and satisfaction with reproductive choices. We started with a focus on searching for three broad themes including: (1) how the CL system affected access to contraception; (2) the desire for children/additional children and whether these desires were affected by CL system contact; and (3) whether/how race and social class were related to #1 and #2. This was followed with deductive coding to condense preliminary concepts and emergent themes into major themes in a second review. This second stage of coding was also based on our quantitative findings. Following discussion between coders, three primary themes emerged which included: (1) judgment and shaming associated with CL histories; (2) internalized guilt; and (3) contraceptive coercion.

## Results

### Surveys

Bivariate analyses (Table 1) indicate that a higher percentage of women with arrest histories (in comparison to those who have never been arrested) have received contraceptive counseling (82.9% versus 68.7%, *p* = 0.008), used one or more methods of long-term contraception (34.2% versus 24.9%, *p* = 0.067), have undergone sterilization (42.7% versus 31.5%, *p* = 0.040), and desired reversal of their sterilization procedure (40.0% versus 9.5%, *p* = 0.00). It is noteworthy that nearly half of women with arrest histories who have had sterilizations would have them reversed if possible.

The fully adjusted logistic regression model for contraceptive counseling is shown in Table 2. We observed that arrest (e^b^ = 2.21, *p* = 0.01) is positively associated with the receipt of contraceptive counseling. Formally, the odds of receiving contraceptive counseling are increased by 2.68 times, holding other variables constant. Lack of insurance was observed to have a negative association with receipt of contraceptive counseling. The remaining variables were not significant.^1^

**Table 2:** Adjusted odds ratios for reproductive outcomes

	Contraceptive Counseling (*n* = 856)		Long-term Contraception (*n* = 856)		Sterilization (*n* = 856)		Desires Sterilization Reversal (*n* = 277)^a^	
	OR	95% CI	OR	95% CI	OR	95% CI	OR	95% CI
Ever Arrested	**2.68**	**1.44, 4.99**	1.35	0.80, 2.29	1.63	0.96, 2.77	**3.87**	**1.30, 11.48**
Age	**0.99**	**0.976, 0.997**	**0.95**	**0.94, 0.97**	**1.05**	**1.03, 1.06**	**0.92**	**0.88, 0.95**
Race (NH White = ref)								
NH Black	0.87	0.53, 1.43	1.00	0.59, 1.70	**1.66**	**1.00, 2.78**	1.63	0.46, 5.75
Hispanic	0.92	0.59, 1.43	**0.61**	**0.38, 0.99**	1.09	0.67, 1.78	2.68	0.86, 8.36
NH Other	0.52	0.25, 1.08	0.47	0.18, 1.19	0.80	0.33, 1.90	1.00	0.07, 14.39
College Degree +	1.31	0.94, 1.84	0.98	0.67, 1.41	**0.68**	**0.47, 0.97**	0.84	0.24, 2.91
Income ($100,000 +)	1.48	0.98, 2.24	0.76	0.49, 1.20	0.84	0.55, 1.28	1.20	0.30, 4.75
Married	1.17	0.84, 1.63	0.94	0.65, 1.35	0.96	0.67, 1.37	0.52	0.19, 1.41
Insurance (private = ref)								
Government	0.93	0.65, 1.34	1.28	0.86, 1.91	1.09	0.74, 1.61	1.17	0.41, 3.28
Other	1.54	0.83, 2.84	1.30	0.73, 2.32	1.20	0.64, 2.24	2.76	0.57, 13.31
None	**0.35**	**0.18, 0.69**	0.92	0.43, 1.98	0.78	0.33, 1.86	0.67	0.07, 6.07
Desired No. Children	1.06	0.93, 1.19	**0.81**	**0.70, 0.93**	**1.19**	**1.04, 1.35**	**1.44**	**1.11, 1.86**
No. of Children	1.00	0.86, 1.16	**1.46**	**1.23, 1.72**	**1.41**	**1.21, 1.66**	0.83	0.53, 1.30
No. Sex Partners	1.03	0.94, 1.12	1.04	0.96, 1.14	1.07	0.99, 1.15	0.99	0.83, 1.18
South	0.90	0.65, 1.24	0.82	0.58, 1.17	1.05	0.74, 1.48	1.64	0.64, 4.18
Constant	3.35	1.73, 6.49	3.15	1.58, 6.32	0.14	0.01, 0.03	3.86	0.33, 45.08

Statistically significant odds ratios boldfaced, *p* ≤ .05

^a^Age at sterilization used in place of age in model predicting reversal of sterilization

The model depicting long-term contraceptive use indicates that once all controls are accounted for, there is not a significant association with arrest history. In models not shown, arrest is positively associated with receipt of sterilization (e^b^ = 1.62, *p* = 0.014); however, the association is diminished to marginal significance with the inclusion of controls (*p* = 0.07) (see Table 2). In the fully adjusted model, a college education or higher was negatively associated with the odds of sterilization; while age, NH Black race, desired number of children, and number of children evidenced positive associations with the receipt of sterilization.

Finally, arrest is significantly associated with desire for reversal of sterilization (e^b^ = 3.87, *p* = 0.02), in the fully adjusted model. Formally, women who have been arrested are more than three times more likely to desire reversal than those who have not been arrested, all else equal.

### Qualitative interviews

Initial coding revealed (see Table 3) that our qualitative interview subjects were diverse in terms of age, race, contraception/fertility history, and criminal legal history. Approximately 40% of our interview subjects identified as more than one race, 20% as Native American, 20% as Asian/Pacific Islander, and 20% as White. The mean age of the sample was 31.3 (range 22–39) at the time of the interview, with an average 2.3 (range 0–5) children, and a mean number of previous arrests of 33.2 (range 1–240^2^). The modal number of previous arrests was five for this sample, with three respondents reporting five prior arrests. Age at first arrest had a wide range (13–23), with a mean of 18.0. Most (90%) of these respondents indicated having a history of substance use disorder. Only one woman was in a romantic relationship at the time of the interview, and 30% reported wanting more children at some point in the future. Two of our interview subjects reported no children and not wanting to ever have children.

**Table 3:** Characteristics of interview participants at the time of the interview (*N* = 10)

Age	**31.3 (5.5)**
Self-reported race	
White	20%
Asian/Pacific Islander	20%
Native American	20%
Mixed/Multiple Races Reported	40%
Highest educational attainment	
Less than high school	20%
High school/GED	60%
Some college	10%
Graduate school	10%
Relationship status	
Single	80%
Unmarried/in a committed relationship	10%
Divorced	10%
Number of children	2.3 (1.6)
Desire for additional children (% yes)	30%
How many more children desired?	0.5 (0.7)
Age at first arrest	18.1 (3.3)
Number of times previously arrested	33.2 (73.6)^a^
History of substance use	90%
Type of previous substance use^b^	
Alcohol	30%
Pain pills	10%
Methamphetamine	80%
Heroin	40%
Marijuana	10%
Methadone	10%
Contraceptive methods ever used^b^	
Tubal ligation	30%
Birth control pills	30%
IUD	30%
NuvaRing	10%
Implanon/Nexplanon	40%
Condoms	60%
Depo-Provera	40%
Emergency contraception	20%
No method	10%
Number of sex partners in the past year	1.3 (1.4)

Numbers reflect percentages or means (with standard deviations in parentheses)

^a^The value shown includes an outlier with a value of 240. Exclusion of this case results in the following values for mean (standard deviation), and range: 10.2 (12.5), 1–40

^b^Percentages sum to more than 100% because more than one category could be selected

Analysis of the 10 qualitative interviews revealed some key themes that speak to and expand on the quantitative survey analysis. These include judgment and shame, internalized guilt, and contraceptive coercion.

#### Judgment and Shaming

Many women in the interview study reported receiving comments from family members and friends such as “…being in that kind of situation, I shouldn’t be having kids” (Lacey^3^) and “I need to be on birth control” (Alexa). Sara reported that her drug counselors, probation officers, and parole officers all commented on how her criminal history and drug use were harming her children. Almost every woman we interviewed described self-judgment and guilt, along with shaming and judgment from others, as important factors affecting future childbearing and decisions about contraception. For example, Christa and Lacey—both of whom who have served time in jail, but not been to prison—described shaming that they received from guards for being pregnant while incarcerated. As Lacey explains:

“So I was using heroin and meth and I got arrested and I was told while I was in jail that I was pregnant. And I was detoxing. I wasn’t using much then but I was detoxing and I had to go to the nursing station because I was pregnant and detoxing. They’re worried about the baby. And I heard some guards making fun of the situation, a thing that—just talking about mothers using while they’re pregnant. And of course in that kind of situation, I can’t retaliate and be like “Well, listen—" Like, I don’t know. I was pregnant first of all. “So please hold your comments to yourself.” You know, I couldn’t say that kind of stuff.”

Christa’s experience was similar to Lacey’s, saying:

“I went into jail one time pregnant, very early stages of pregnancy, and you kind of get berated in talked down to by the C.O.s [Corrections Officers] and stuff telling you “Oh, so now, you’re gonna have kids and stuff and that poor kid is gonna grow up with a delinquent mom and all that.”… And so, I mean that wasn’t very helpful being pregnant and then, having the stress of that and the way they talk to you about it makes you feel like shit and stuff is not very productive in having a healthy pregnancy.”

Later on, Christa added that she heard comments from C.O.s “…that we shouldn’t have kids. We shouldn’t be allowed if we have a criminal history of that because we’re bad for stability for our kids.”

Meanwhile Alexa—who spent time incarcerated in a juvenile detention center, but never in an adult prison—explained “so I actually used while I was pregnant…with 2 out of 4 children, so I would get a lot of, you know, discrimination and judgments and people telling me that I didn’t need to have any more kids, that I needed to be on birth control. People telling me I’m a shitty parent which, given you know, I guess it's a shitty choice, but when you're [in the midst of] addiction…”.

#### Internalized guilt

Most of the women we interviewed internalized the judgment and shaming they experienced, which translated into feelings of guilt surrounding their fertility decisions. Carla (age 39) reported being arrested only once in her lifetime, and waiting in jail for her trial for almost two years before being sent to prison. She said she felt guilty about wanting to have more kids after her release because she worried her daughter will be upset if she sees her mom being there for the new kid when she *wasn’t* there for her daughter due to substance use and incarceration. As Carla explains, while she was incarcerated her phone calls with her daughter often ended up with her daughter questioning: “Well, why haven’t you been around? Do you just not love me?” And Carla went on to say:

“And it’s like, what do you say? You know, how do you answer that when you’ve been ripped away from your kid because the corrections, you know, the prison system has done that … That was a lot of years I missed, you know, basically, her whole life growing up …”

Later on, Carla continued:

“I started to feel some guilt around I don’t know if I should have more kids. And my daughter, how’s that gonna make her feel that I’m gonna be there for this new child but I wasn’t there for her?”

#### Coercion and lack of agency in birth control

A final important theme we identified was the role coercion played in women’s decisions regarding contraception or method selection. Three of the 10 participants mentioned being forced or coerced by various agents in the CL and health system when accessing contraception. One example is Carla, who felt “pressured” to get a contraceptive implant in her arm while she was incarcerated. Carla said that a nurse in prison told her that since she was due to be released it was “time [to] schedule you for getting birth control.” Carla was confused since she thought, “but I’m not sexually active” and proceeded to ask questions about why an implant was being encouraged. She was told “Well, you should do this one.” Despite Carla’s requests for more information, the nurse followed up with “’I’m just gonna go ahead and get you scheduled for it.’ And she rushed out of the room.”.

Another respondent, Christa, who had served several probation and jail sentences, said after she was found, at age 15, breaking into a home in search of food and heat, she became a ward of the state. The state then put Christa on Depo-Provera and gave her no choice. In her words, “…the state put me on it [Depo-Provera]…I had no options.”

Alexa, a single mother of 4 who identified as Native American and White, similarly felt like she had no options when “each time that I’ve had a child it [birth control] is kind of brought up and kind of almost forced upon me in a sense…they say a 100 times, you know, if I want to start thinking about birth control.” Alexa went on to explain that she almost didn’t want to go back for her next checkup “because I am not ready to decide…[and] I don’t want to be looked at [as though] I need to be on it…and I don’t want to be looked at [like] I just want to …start an army.”

Although she didn’t describe her tubal ligation as coercive, Sara, a 33 year-old Asian women who had served time in both state and federal prison for drug-related crimes, described some pressure, explaining that when she was younger (22), and after she had given birth to her third child, “the doctor had asked if I wanted to do it [tubal ligation] and I did do it.” She went on to say “I mean I kinda regret it actually since I was so young, but…” Sara indicates that she might like to have this process reversed one day to have another child.

### Qualitative interviews summary

In sum, the qualitative interviews we conducted shed some light on how to interpret the quantitative survey results. Our analysis suggests that women who have histories of CL contact encounter a great deal of judgment and shaming surrounding their pregnancies or desire for children via numerous agents of social control. The women we interviewed detailed experiences with probation and parole officers, drug counselors, prison/jail guards, doctors and nurses, and family and friends who felt free to comment on their fertility desires and/or behavior, and often explicitly suggested they should not have children or were unfit to have the ones they did (as in COs stating that women with a criminal history shouldn’t be allowed to have kids). Additionally, CL-involved women commonly struggle with guilt over the perceived harm they have done to their children, which can be internalized as feelings of being underserving or faulty for desiring more children. Many of our interviewees made statements suggesting they felt they deserved the judgment and didn’t deserve to have children (i.e., Lacey felt unable to defend herself, Christa didn’t reject the delinquent mom label, and Alexa accepted the judgment that she probably shouldn’t have more kids). Some women attempted to push back against these feelings of guilt by focusing on doing things right the next time with another/new child. But, the more common response was to accept these judgments regarding their fitness, or lack thereof, as mothers. As a result of these shaming processes, these women tend to embrace long-acting forms of contraception that may provide breathing room from their “chaotic” life or give them some sense of agency. A relatively small number of women (three out of ten) recounted experiences of coercion related to contraception, yet these were serious enough to lead women to feel that various forms have been forced on them. An additional woman described being prompted to undergo tubal ligation and the desire for reversal of that procedure. This lack of choice in their reproductive health care seems to lead to frustration, anxiety, and—for some—the receipt of unwanted methods with long-term or permanent implications.

## Discussion

In this study, we endeavored to address whether CL system contact (arrest) shapes women’s reproductive capacity, i.e., receipt of contraceptive counseling, long-term contraceptive use, receipt of sterilization, and desire for sterilization reversal; and whether contact with the CL system contributes to reduced reproductive autonomy. Our study reveals several important findings. First, our survey results revealed that arrest is significantly associated with several reproductive outcomes including receipt of contraceptive counseling, receipt of sterilization (prior to the inclusion of covariates), and desire for reversal of sterilization. This study advances knowledge because relatively few prior studies have focused on arrest as a factor in reproductive autonomy, despite evidence of racial disparities that produce disadvantage (Spinney et al., 2018). Arrest is also important to consider because comparatively few women go on to experience imprisonment (Gartner, 2011), a finding that was also demonstrated in our qualitative interview sample. Indeed, prior work has shown that long-term or permanent contraception is utilized as a means through which to *avoid* incarceration. Our findings are in line with this assertion and underscore how even the earliest stage of contact with the CL system may impact women’s reproductive outcomes.

Notably, we observed that despite controls for social disadvantage, women with arrest histories were marginally more likely to undergo sterilization and significantly more likely to desire reversal of their sterilization procedures. This is consistent with prior work indicating higher rates of sterilization in CL system-involved women (Pruitt et al., 2010; Ramaswamy & Kelly, 2014). Researchers (e.g., Ramaswamy & Kelly, 2014) have speculated that these women may opt for sterilization because of financial constraints, encouragement from providers and family members, or familiarity with the procedure (i.e., family members who had one). Our qualitative work would seem to support at least some of these findings, especially the concerns about financial or other social constraints, and encouragement or coercion from family, friends, and other service providers.

A second contribution of this study comes from our ability to offer several additional explanations for why arrest might be expected to pattern reproductive outcomes. Arrest brings women into contact with various agents connected to the criminal legal system who may attempt to influence or even force these women’s reproductive decisions. For instance, a person arrested as a juvenile recalled how her arrest led to involuntary use of an injectable form of birth control. Arrests that do not result in time spent in jails or prisons may lead to probation, which offers another avenue through which CL agents may influence reproductive decisions as women may feel compelled to utilize undesired methods to show good faith or efforts toward rehabilitation.

In addition, arrest histories frequently dovetail with substance use—90% of our sample reported substance use histories—and patterns of addiction seem to relate directly to women’s feelings about their childbearing. These women, many of whom were diverted out of the CL system and into treatment, received consistent messages shaming them for their addiction and, for some, using while pregnant. The overwhelming message is that they were “shitty mothers”, which clearly factored into decision-making regarding future childbearing and contraceptive choices. Thus, fertility limitation may occur via processes that lead women to devalue themselves and their own childbearing. In other words, it is possible that women with CL contact may have internalized views suggesting that they are unfit mothers and may adjust their fertility goals and contraceptive behaviors in accordance with this view (Geiger & Fischer, 2005; Thompson & Newell, 2021).

Finally, we observed that women with arrest histories are more likely to have received contraceptive counseling. This may suggest that health professionals contribute to these outcomes. For instance, there is reason to believe health professionals are aware of prior substance use as this information is collected during well-woman visits. At least one of our interview respondents explained volunteering her criminal history to her (outside of prison) gynecologist and several others hinted that this kind of disclosure was common. Prior studies have shown that bias on the part of health professionals leads to differential treatment and recommendations (Binswanger et al., 2011; Downing, et al., 2007; Spencer & Grace, 2016), and value judgments surrounding criminal histories, substance use, and social marginality may lead to encouragement to use long-term or permanent methods of contraception (Higgins et al., 2016; Ramaswamy & Kelly, 2014; Yee & Simon, 2011). In addition, recent efforts to facilitate continuity of care across systems using electronic health records (Freudenber & Heller, 2016) may increase the opportunities for health providers to learn of previous substance use, CL involvement, and incarceration, and is worthy of additional study. The possibility that women face coercion both in and out of the CL system, first via the efforts of CL agents and later by health providers, is noteworthy and future research should explore how arrest histories may doubly burden these women.

Limitations with respect to this study include the cross-sectional nature of the quantitative data as well as sampling limitations for the qualitative interviews. Because we rely on cross-sectional data to model our outcomes, we are unable to ensure causality with respect to observed associations. For example, it is possible that some reproductive outcomes may have occurred prior to arrest. Future research should utilize longitudinal data to account for this possibility. However, our findings are bolstered by women’s interview accounts, which suggest that at least some instances of coercion occurred post-arrest. While efforts were made to capture the responses of women with varied sociodemographic characteristics, the purposive nature of the sampling strategy, along with COVID-19 limitations, resulted in a small interview sample and no Black respondents (although there were several women who identified as “mixed race” or “multiple races,” which did include Black respondents). Additional research is needed to capture the experiences of a larger and more diverse group of women. Also, given the qualitative nature of the interview data, results may not be generalizable to larger populations. With respect to the quantitative data, we were unable to directly account for substance use. Given its prevalence among interviewed women, future studies should examine its role as it seems to be an important mechanism that may underlie observed associations. In addition, we were unable to incorporate additional measures of CLS involvement (e.g., recent arrest, incarceration) due to small cell sizes. Future studies should examine whether there are differences in the experiences of women who have had varying levels of contact with the CL system. Nonetheless, the use of nationally representative survey data in combination with in-depth interviews is a notable strength, and this study yields important insights into the ways in which CL contact may translate into undesirable reproductive outcomes.

## Conclusions

Overall, our findings suggest a mismatch between desired reproductive outcomes and actual experiences among women with arrest histories. As a first step to addressing this mismatch, greater investments should be made in expanding access to an array of non-permanent contraceptive options to women who interact with the criminal legal system. Our qualitative findings highlight the efforts of actors within and outside the CL system to regulate the reproduction of CL-involved women via judgment, coercion, and a general lack of sensitivity. Indeed, early CL contact appears to provide entree for a host of individuals to weigh in on their reproduction. These women’s experiences are underscored by greater odds of contraceptive counseling and desire for reversal of sterilization among women with arrest histories in our quantitative study. To encourage greater sensitivity among various service providers for CL-involved women, programs designed to support those caring for at-risk populations (Caring with Compassion Curriculum (ACP, 2023)) or to improve health care quality and communication (e.g., Institute for Healthcare Improvement Toolkit (IHI, 2023)) could be adapted for use with these populations. CL-involved women could potentially benefit from support and peer advocacy programs, such as the YWCA’s Family Preservation Project (FPP) in Oregon which “promotes both individual and system level change to reduce the collateral consequences of parental incarceration” by providing resources both during incarceration and peer advocacy for those reentering the community (https://www.ywcapdx.org/family-preservation-project); or those that increase their health literacy and engagement (e.g., Ask Me 3 (IHI, 2023); Be Prepared to Be Engaged (AHRQ, 2018)).

As a specific example of how peer advocacy programs can have beneficial impacts, Carla described her experiences with the Family Preservation Project (FPP) building her up, compared to the traditional prison/CL system tearing her down:

“…prior to FPP, the way that the prison system, you know, they don’t build you up. They, like really tear you down. Like, 10 days prior to my release, the captain of the minimum side pulled me into her office and told me that she would see me back and that this time, I would never leave prison …she said all of those things, I was gonna come back to prison and that I would die in there and so, the prison system, without FPP being there to like support me and talk me through that stuff, things like that from that staff member would have made me feel like I’m not gonna be able to do anything when I get out of prison. Like, I’m garbage, I’m just an inmate. I’m just a criminal but the FPP program was one of the huge, you know, pieces that were always there, always supportive. Cuz they’re onsite at the prison and then, all of my mentors that I had and my family to help me move past that but most women in there, they don’t have a support system. They don’t have family. Like, I was blessed to be able to have family that were there with me the whole time. They don’t have that, so, that’s all they hear and when you keep hearing that over and over, and over again, you start to believe it even though you know this isn’t true but you – you can’t help but start to believe it because that’s all you’re being told that’s all you’re being fed.”

In our view, the CL system should be deemphasized as a site through which to offer reproductive care given historical and contemporary patterns of exploitation. Furthermore, additional efforts are needed to encourage sensitivity and awareness of the political and historical context among actors in and around the CL system. Taken together, our findings suggest the need for careful attention to how exposure to the CL system contributes to fertility limitation among affected women.

## Data Availability

Interview data not available due to ethical restrictions. Due to the nature of this research, participants of this study did not agree for their data to be shared publicly, so supporting data is not available. The survey data generated and/or analyzed during the current study are not publicly available due to data held in a private repository. Raw data were generated at NORC. Outputs supporting the findings of this study are available from the corresponding author [GG] on request.

## Appendix

### Qualitative interview guide

#### Demographic/background questions

1. What do you consider to be your race?
2. How old are you?
3. How far did you go in school?
4. What is your marital/relationship status?
5. How many children do you currently have? How many do you (eventually) want to have?
  - (if any additional children are desired): when do you want to have these children?

#### Criminal history/criminal justice experience

6. Criminal history:
  - How many times have you been arrested?
    - Follow up questions:
    - i. How old were you the 1st time you were arrested?
    - ii. What happened after your previous arrests? (did you spend any time in jail (prior to a conviction) after your arrest? Did you have to pay bail? Were you eventually convicted (or did you plead guilty)? Were you sentenced to probation, jail, or prison?)
  - (if not answered above): Have you ever been convicted of a crime? (if so, what was it?/what was/were the crime(s)?)
  - (if not answered above): Have you ever been incarcerated? (if so, was it prison or jail? How long were you there?)
  - Have you ever had a substance abuse problem? (if so, what substance(s) did you have a problem with?)

7. How has your criminal justice experience affected your relationships with your child/children? Has this experience impacted your decisions regarding future childbearing?

#### Contraception use, knowledge and experience

8. How many sex partners have you had within the last year?
9. What is your contraception history (i.e., which forms have you used, and when?) [probe/provide examples: no method, condoms, birth control pill, injectable (ex. Depo), implant (ex. Norplant), IUD (ex. Mirena, Paragard), rhythm/withdrawal, emergency contraception, female/tubal sterilization, male vasectomy, other.]
  - How did you make decisions about birth control? [probe: Where/how did you learn about your birth control options?]
    i. Follow up question: Do you feel differently about any of these birth control decisions now? (why/why not?)
  - Where do you usually go to receive women’s health services (birth control, STD screenings, PAP smear, etc.)? [probe/provide examples: private office, public clinic, hospital clinic or emergency room, urgent care, don’t have a usual place]
    i. Follow up question: When you go to these places [private office, public clinic, hospital clinic or emergency room, etc.] what kinds of services do you seek? (birth control, STD and/or HPV screenings, PAP smears, pregnancy/childbirth services, etc.)

10. What is your preferred method of contraception (if there is one)?
  - How often do you use this method or methods?
    - Follow up question: What weighs in on that decision?

11. Have the following people talked to you about contraception and/or whether you want to have children? (for each, if respondent says ‘yes’ follow up with questions about who initiated the conversation, if they commented on desire to have kids, how many birth control options were discussed, whether they ever felt pressured to use a particular method, and whether the respondent felt they were listened to/respected in the decision-making process):
  - Doctors (primary care, gynecologists, ER physicians, jail/prison physicians, others)
  - Nurses
  - Parole/probation officers
  - Social workers
  - Teachers
  - Police officers
  - Guards in jail/prison
  - Judges
  - Attorneys (either defense attorneys or prosecutors)
  - Any other worker in the criminal justice system
  - Another prison/jail inmate

#### Social class, race and contraception access

12. Are you able to access contraceptives when you request/want them?
  - Why or why not?

13. Have you ever been offered or encouraged to consider free or low-cost birth control? If so, who offered it/initially mentioned it?
  - Doctors (primary care, gynecologists, ER physicians, jail/prison physicians, others)
  - Nurses
  - Parole/probation officers
  - Social workers
  - Teachers
  - Police officers
  - Guards in jail/prison
  - Judges
  - Attorneys (either defense attorneys or prosecutors)
  - Any other worker in the criminal justice system
  - Another prison/jail inmate

14. Do you think your arrest/substance use/criminal justice history has ever affected advice you were given about birth control? (if yes, describe a time/who was involved, etc.)
  - follow up if respondent seems stuck/asks for clarification of what this question means: for example, has a judge ever expressed an opinion about whether you should have children? Has anyone ever commented on your drug use or arrest history and how it impacts children? Has anyone suggested that you shouldn’t have (any/additional) children because of these issues?

15. Do you think your own race has ever affected advice you were given about birth control (including the advice potentially given above, in response to question 12)? (if yes, describe a time/who was involved, etc.)
  - follow up to this question: Do you believe that the race of actors in/around the criminal justice system (including the following list of actors) affected your thinking on these reproductive/contraceptive decisions? (if yes, follow up with how?/Why?)
  - probes (if respondent has difficultly coming up with an answer): (a) can you think of any examples you’ve encountered of woman of color being treated differently in the criminal justice system and/or regarding contraception? (b) [if still nothing] Can you hypothetically think how race could/may play a role in these issues?
    i. Doctors (primary care, gynecologists, ER physicians, jail/prison physicians, others)
    ii. Nurses
    iii. Parole/probation officers
    iv. Social workers
    v. Teachers
    vi. Police officers
    vii. Guards in jail/prison
    viii. Judges
    ix. Attorneys (either defense attorneys or prosecutors)
    x. Any other worker in the criminal justice system
    xi. Another prison/jail inmate

16. Has your experience with contraception been affected by COVID-19 and “social distancing” associated with the coronavirus? (Has your access to medical care/contraception changed? What about your thoughts about fertility/family planning?)

17. Anything else to add on these topics that I didn’t ask about? Anything else it would be important for us to know?

## Footnotes

1 In analyses not shown, we replaced our dichotomous measure of income with an expanded version to account for the possibility that poverty might explain some of the observed associations. However, the use of more refined measures of income did not appreciably affect any of the model outcomes.

2 Excluding the outlier (respondent with 240 self-reported arrests), the range was 1–40 and mean was 10.22.

3 All names are pseudonyms.
